# The association between leptin and inflammatory markers with obesity indices in Zanzibari children, adolescents, and adults

**DOI:** 10.1002/osp4.466

**Published:** 2020-11-04

**Authors:** Maria Adam Nyangasa, Christoph Buck, Soerge Kelm, Mohammed Ali Sheikh, Kathrin Günther, Antje Hebestreit

**Affiliations:** ^1^ Leibniz Institute for Prevention Research and Epidemiology—BIPS Bremen Germany; ^2^ Centre for Biomolecular Interactions Bremen Faculty for Biology and Chemistry University Bremen Bremen Germany; ^3^ Environmental Analytical Chemistry and Eco‐toxicology Lab State University of Zanzibar Zanzibar Tanzania

**Keywords:** inflammatory markers, leptin, obesity indices, sub‐Sahara Africa

## Abstract

**Background:**

Research from Western populations describes abdominal obesity as a low‐grade inflammatory disease; less is known from tropical areas with high pathogen burden.

**Objectives:**

This cross‐sectional study investigated whether obesity contributes to low‐grade inflammation in 587 individuals from randomly selected households in Zanzibar.

**Materials and Methods:**

The Association between obesity indices (body mass index [BMI], waist circumference [WC], and percentage body fat [%BF]), leptin, and inflammatory markers (C‐reactive protein [CRP], interleukin‐6 [IL‐6] and tumor‐necrosis factor‐α [TNF‐α]) was investigated using multinomial logistic regression analysis, accounting for ordinal outcome variables with four categories; 1st–4th quartile.

**Results:**

Study participants were between 5 and 95 years; 49.6% were male. Mean serum levels were; leptin: 4.3 ± 5.2 ng/ml, CRP: 0.19 ± 0.42 µg/ml, IL‐6: 2.8 ± 5 pg/ml, and TNF‐α: 5.3 ± 5.2 pg/ml. Obesity indices were associated with leptin and CRP in the third and fourth quartiles in single models. In combined models, associations were observed between BMI (OR = 6.36 [95% CI, 1.09; 34.12]); WC (OR = 4.87 [95% CI, 1.59; 14.94]); and %BF (OR = 19.23 [95% CI, 4.70; 78.66]) and leptin in the fourth quartile; also between %BF and CRP in the third quartile (OR = 3.49 [95% CI 1.31; 9.31]).

**Conclusion:**

Total body fat was associated with low‐grade inflammation in this tropical population rather than body fat distribution such as abdominal obesity. This may increase the risk of insulin resistance and other obesity‐related metabolic and cardiovascular health endpoints.

## 1 INTRODUCTION

Obesity has been associated with increased risk of developing chronic inflammatory diseases and type 2 diabetes mellitus^1^ and is characterized by excessive or abnormal body fat in the adipose tissue. Adipose tissue stores triglycerides and produces adipokines by acting as an endocrine organ,^2^ which plays an important role in controlling appetite, lipid metabolism, and insulin resistance.^3^ In individuals with obesity, adipocytes are enlarged and their secretory profiles are altered.^4^ They produce hormones such as leptin and generate increased amounts of proinflammatory cytokines, which, among others, include interleukin‐6 (IL‐6) and tumor‐necrosis factor (TNF‐α).^5^ IL‐6 and TNF‐α stimulate the production of C‐reactive protein (CRP) that is generated in the liver.^1^ For example, IL‐6 is released by the visceral adipose tissue into the portal circulation^3^ and is elevated in patients with lipid abnormalities and insulin resistance. Similarly, TNF‐α is a proinflammatory cytokine that exerts lipid metabolism and insulin signaling in adipose tissue, thus its levels are elevated in individuals with obesity and reduced with weight loss.^5^ CRP has been used as a marker for obesity‐related low‐grade inflammation, which contributes to insulin resistance.^1^ Furthermore, higher levels of inflammatory biomarkers CRP, IL‐6, and TNF‐α were reported to be associated with increased glycated hemoglobin (HbA1c) as a marker for type 2 diabetes mellitus.^6^ In turn, obesity measured using different indices, such as waist circumference (WC) and body mass index (BMI), was associated with higher serum CRP levels and TNF‐α.^3^, ^7^

Very few studies in sub‐Saharan Africa have investigated the association of leptin and inflammatory markers with obesity indices,^8^, ^9^ and none of them explicitly investigated the role of obesity in low‐grade inflammation in a Zanzibari population, whose obesity prevalence and the associated comorbidities have increased over the years.^10^ The hypothesis of this study is that individuals with obesity are more likely to have elevated leptin levels and inflammatory markers (including CRP, IL‐6, TNF‐α). To this end, the weight status of Zanzibari children, adolescents, and adults was assessed, depending on their serum levels of leptin, CRP, IL‐6, TNF‐α, and IL‐8. First, the correlation of leptin and inflammatory markers (CRP, IL‐6, TNF‐α) was analyzed. In a second step, a quasi‐linear association of obesity indices with leptin, CRP, IL‐6, and TNF‐α was investigated.

## 2 MATERIALS AND METHODS

### 2.1 Study area, study design, and sampling

Zanzibar Island is located approximately 25 km off the coast of Mainland Tanzania. It comprises two main Islands, Unguja and Pemba, and has a population of 1.3 million people, almost 63% of whom live in Unguja.^11^ Administratively, Zanzibar is divided into five regions, three in Unguja and two in Pemba. Each region has two districts and each district is subdivided into smaller administrative units, known as Shehias (wards).

Participants for this cross‐sectional study were randomly selected in 2013, in a representative population in Unguja Island, with households serving as sampling units.^12^ The original study was powered to estimate the prevalence of malnutrition in the Zanzibari population, including possible correlates as described elsewhere.^12^ For the present study, a sub‐sample of participants fulfilling the study inclusion criteria (age, sex, height, and weight), anthropometric measurements, and provided complete blood samples for leptin and the inflammatory markers (above 5 years) were included. All persons living in the same household were enrolled in the study, irrespective of their age or gender. This was done to help account for inequality aspects. Zanzibari mostly live in extended families, and some families had up to four generations living together in one household, thus providing a large age range. The study was performed according to the Helsinki Declaration and the study protocol was evaluated and approved by the Ethics Committees of the University of Bremen and of the Zanzibar Ministry of Health and the Zanzibar Medical Research and Ethics Committee. All participants above 16 years gave a written consent and parents/guardians gave a written consent on behalf of their children who were below 16 years.

### 2.2 Socio‐economic and demographic indicators

Socio‐economic and demographic indicators were assessed at household level.^13^ The highest education level of the head of the household was assessed using the International Standard Classification of Education^14^ and was categorized as low education level and high education level. The area of residence was recorded and categorized as urban and rural area. As malnutrition is an important factor in this association,^15^ the Individual Dietary Diversity Score (IDDS) was calculated based on 14‐food groups recommended by the Food and Agriculture Organization,^16^ and included as a confounder. Since the median of IDDS in this study was 4, two categories were then developed according to the median; low ≤4 (consumption of less than four food groups) and high >4 (consumption of more than four food groups). All questionnaires were developed in English, translated into Swahili and then back‐translated, to check for translation errors.

### 2.3 Inclusion and exclusion criteria

In total, 616 participants provided complete blood samples for the analysis of leptin, TNF‐α, IL‐6, IL‐8, and CRP. Participants with serum levels outside the detection limits for leptin and each inflammatory marker were excluded. For the overall study analysis, 587 participants additionally fulfilled the inclusion criteria (availability of data on age, sex, weight, and height) and provided complete covariate information (area of residence, education level, and IDDS). Of these, data from 71 participants were excluded due to pregnancy, nonfasting status, and medication use. All individuals with a high CRP concentration >10 µg/ml (*N* = 8) were excluded as an acute inflammation was presumed. A further *N* = 27 individuals in the highest quartiles of IL‐8 as well as CRP were removed from the descriptive analysis as this indicated an acute infection. As extremely high IL‐8 levels were observed in many individuals, the variable IL‐8 was excluded from the final analysis. Four complete data sets were created: one for leptin and three for each of the inflammatory markers. In order to exclude individuals with a possible acute infection not related to obesity, we excluded 10% of the top extreme values in each data set, thus the final sample sizes included in the final analyses were as follows: CRP = 509; IL‐6 = 447; TNF‐α = 429; and Leptin = 465.

### 2.4 Obesity indices

Bodyweight, body fat percentage (using bioelectrical impedance analysis), height and WC were measured.^12^ For children and adolescents, the BMI was calculated as kg/m^2^ and then transformed to age‐ and sex‐specific z‐score and percentiles, as well as categories for overweight (BMI between >75th and <95th percentile) and obesity (BMI >95th percentile) according to the WHO centile curves.^17^, ^18^ For adults, overweight/obesity was defined as BMI ≥25 kg/m^2^ as recommended by the WHO.^19^ High WC was defined as WC ≥ 90th percentile for children below 10 years^20^ and for adolescents below 16 years.^21^ For those above 16 years, high WC was defined as WC > 94 cm for males and >80 cm for females, as recommended by the International Diabetes Federation.^21^ High percentage body fat (%BF) for participants below 18 years was categorized as ≥85th percentile.^22^ For those above 18 years, high %BF was categorized as ≥20 for men and ≥32 for women.^23^

### 2.5 Leptin and inflammatory markers

Venous blood was collected from eligible participants in an overnight fasting status. From each participant, 10 ml (6 ml for children) of venous blood was collected in a clot activator vacutainer PET tube (BD, 367896 [10 ml] and BD 368815 [6 ml]) and kept at 4°C until serum was prepared by centrifugation on the same day.^12^ Leptin, CRP, IL‐6, IL‐8, and TNF‐α were detected using enzyme‐linked immunosorbent assay kits (Human Leptin: K15164C; Human CRP kit: K151EPC; Human cytokines: customized kit (7‐spot) from Meso Scale Discovery (Rockville) on a SECTOR Imager 2400A, applying the Discovery Workbench software package (version 4.0). Detection ranges were 10^2^–5 × 10^4^ pg/ml (leptin), 2 × 10^−2^–2 × 10 μg/ml (CRP), 2 × 10^−1^–6 × 10^2^ pg/ml (IL‐6), 1 × 10^−1^–5 × 10^2^ pg/ml (IL‐8), and 3 × 10^−1^–3 × 10^2^ pg/ml (TNF‐α).

### 2.6 Statistical analysis

Statistical analysis was performed using SAS 9.3 (SAS Institute). Mean and standard deviation for continuous variables were calculated, stratified by weight status. Leptin, CRP, IL6, and TNF‐α levels were categorized using sex‐ and age‐group‐specific quartile ranges (Q1–Q4). For obesity indices, very severe thinness and severe thinness were merged into one category (severe thinness, Table 2.) and obesity classes I, II, and III were merged into one category (obesity, Table 2). For the analysis of the statistical models (Tables 4 and 5), two categories for each obesity marker that is BMI (overweight/obesity vs. normal weight), WC (high vs. low), and %BF (high vs. low) using sex‐ and age‐specific cut‐offs, were created. Pearson's correlation coefficient was calculated to test for intercorrelation. Associations between obesity indices and quartiles of leptin and the inflammatory markers were modeled using multinomial logistic regression analysis. This was based on the LOGISTIC procedure that accounted for ordinal outcome variables with four categories (Q1–Q4), with Q1 serving as the reference variable. Regression analysis was conducted in two steps: (1) 12 models regressing three obesity indices on leptin and each of the three inflammatory markers, (2) Since BMI, WC, and %BF are interrelated, their predictive power on leptin and the three inflammatory markers was investigated in a regression model in which all obesity indices were regressed on each of the outcome variables. Thus four models were regressed for leptin and each of the three inflammatory markers with all the three obesity indices combined in one model. Sex, age range, education level, area of residence, and IDDS categories were included in all multinomial logistic regression analysis models as adjustment variables.

**TABLE 1:** Descriptive statistics of anthropometric, biochemical variables, and IDDS of the study participants stratified by sex in terms of mean, SD

	Underweight/Normal weight			Overweight/Obesity			All		
	*N*	Mean ± SD	Range	*N*	Mean ± SD	Range	*N*	Mean ± SD	Range
Age (y)	360	24 ± 16	(4.9, 91)	129	42 ± 17	(8.6, 81)	489	29 ± 18	(4.9, 91)
Height (cm)	360	154 ± 16	(108, 190)	129	160 ± 9.8	(127, 185)	489	155 ± 15	(108, 190)
Weight (kg)	360	46 ± 15	(15, 80)	129	74 ± 13	(29, 116)	489	53 ± 19	(15, 116)
BMI (kg/m^2^)	360	19 ± 3.4	(11, 25)	129	29 ± 4.0	(18, 49)	489	21 ± 5.7	(11, 49)
WC (cm)	360	68 ± 12	(12, 96)	129	93 ± 12	(61, 129)	489	74 ± 16	(12, 129)
Body fat (%)	360	17 ± 8.1	(1.6, 53)	129	33 ± 8.4	(9.6, 53)	489	21 ± 11	(1.6, 53)
Leptin (pg/ml)	355	3.5 ± 4.7	(0.05, 21)	85	7.9 ± 5.9	(0.29, 21)	440	4.3 ± 5.2	(0.05, 21)
CRP (µg/ml)	356	0.14 ± 0.4	(0.00, 3.4)	126	0.33 ± 0.6	(0.00, 3.5)	482	0.19 ± 0.4	(0.00, 3.5)
IL‐6 (pg/ml)	311	2.9 ± 5.1	(0.09, 29)	109	2.6 ± 4.9	(0.04, 28)	420	2.8 ± 5.0	(0.04, 29)
IL‐8 (pg/ml)	293	285 ± 311	(3.2, 1E3)	103	222 ± 301	(2.7, 1E3)	396	269 ± 309	(2.7, 1E3)
TNF‐α (pg/ml)	302	5.5 ± 5.3	(1.1, 24)	106	4.7 ± 4.7	(0.99, 24)	408	5.3 ± 5.2	(0.99, 24)
IDDS	360	4.7 ± 1.1	(2.0, 9.0)	129	4.5 ± 1.2	(2.0, 8.0)	489	4.6 ± 1.1	(2.0, 9.0)

Abbreviations: CRP, C‐reactive protein; IDDS, individual dietary diversity score; IL‐6, interleukin‐6; IL‐8, interleukin‐8; SD, standard deviation; TNF‐α, tumor necrosis factor; WC, waist circumference.

**TABLE 2:** Characteristics of the study population (*N* = 359)

		Severe thinness		Thinness		Normal weight		Overweight		Obese		All	
		*N*	%	*N*	%	*N*	%	*N*	%	*N*	%	*N*	%
All		52	100	62	100	177	100	55	100	13	100	359	100
Socio‐demographic information													
Gender													
Male		30	57.7	32	51.6	79	44.6	30	54.5	7	53.8	178	49.6
Female		22	42.3	30	48.4	98	55.4	25	45.5	6	46.2	181	50.4
Age range													
5–18		37	71.2	43	69.4	66	37.3	4	7.27	0	0.00	150	41.8
18–45		10	19.2	14	22.6	73	41.2	27	49.1	8	61.5	132	36.8
45+		5	9.62	5	8.06	38	21.5	24	43.6	5	38.5	77	21.4
Educational level													
Low		43	82.7	36	58.1	80	45.2	24	43.6	5	38.5	188	52.4
High		9	17.3	26	41.9	97	54.8	31	56.4	8	61.5	171	47.6
Area of residence													
Rural		15	28.8	19	30.6	52	29.4	12	21.8	5	38.5	103	28.7
Urban		37	71.2	43	69.4	125	70.6	43	78.2	8	61.5	256	71.3
Leptin and inflammatory markers in quartiles													
Leptin													
First quartile		28	53.8	17	27.4	50	28.2	2	3.64	0.00	0.00	97	27.0
Second quartile		12	23.1	26	41.9	40	22.6	11	20.0	1	7.69	90	25.1
Third quartile		7	13.5	12	19.4	47	26.6	18	32.7	5	38.5	89	24.8
Fourth quartile		5	9.62	7	11.3	40	22.6	24	43.6	7	53.8	83	23.1
CRP													
First quartile		18	34.6	21	33.9	50	28.2	12	21.8	1	7.69	102	28.4
Second quartile		13	25.0	14	22.6	49	27.7	14	25.5	3	23.1	93	25.9
Third quartile		12	23.1	17	27.4	50	28.2	17	30.9	3	23.1	99	27.6
Fourth quartile		9	17.3	10	16.1	28	15.8	12	21.8	6	46.2	65	18.1
IL‐6													
First quartile		11	21.2	14	22.6	54	30.5	23	41.8	2	15.4	104	29.0
Second quartile		15	28.8	15	24.2	49	27.7	13	23.6	3	23.1	95	26.5
Third quartile		18	34.6	18	29.0	41	23.2	12	21.8	7	53.8	96	26.7
Fourth quartile		8	15.4	15	24.2	33	18.6	7	12.7	1	7.69	64	17.8
TNF‐α													
First quartile		6	11.5	22	35.5	52	29.4	13	23.6	2	15.4	95	26.5
Second quartile		17	32.7	10	16.1	45	25.4	19	34.5	5	38.5	96	26.7
Third quartile		18	34.6	14	22.6	40	22.6	16	29.1	3	23.1	91	25.3
Fourth quartile		11	21.2	16	25.8	40	22.6	7	12.7	3	23.1	77	21.4
IDDS													
IDDS category													
Low	20		38.5	30	48.4	73	41.2	22	40.0	7	53.8	152	42.3
High	32		61.5	32	51.6	104	58.8	33	60.0	6	46.2	207	57.7

Abbreviations: CRP, C‐reactive protein; IDDS, individual dietary diversity score; IL‐6, interleukin‐6; TNF‐α, tumor necrosis factor.

**TABLE 3:** Correlation between leptin and inflammatory markers

Leptin and inflammatory markers	Pearson correlation coefficients			
	Prob > |r| under H0: Rho = 0			
	Number of observations			
	CRP	IL‐6	TNF‐α	Leptin
CRP	1.00	−0.065	−0.065	**0.099**
		0.19	0.19	**0.04**
	482	414	404	**434**
IL‐6		1.00	**0.764**	−0.022
			**<0.0001**	0.67
		420	**400**	381
TNF‐α			1.00	−0.01717
				0.7424
			408	369
Leptin				1.00
				440

*Note*: The bold values indicate statistically significant (*p*‐value < 0.05).

Abbreviations: CRP, C‐reactive protein; IL‐6, interleukin‐6; TNF‐α, tumor necrosis factor.

## 3 RESULTS

### 3.1 Study characteristics

The study participants were between 5 and 95 years old, with the largest proportion of individuals being between ≥5 and <18 years (42%) (Table 1). The mean concentrations for leptin and CRP were higher in participants with overweight/obesity compared to participants who were underweight/normal weight (7.9 ± 5.9 ng/ml vs. 3.5 ± 4.7 ng/ml and 0.33 ± 0.6 µg/ml vs. 0.14 ± 0.4 µg/ml, respectively). On the contrary, the mean concentration values for the inflammatory markers IL‐6, IL‐8, and TNF‐α were slightly higher in underweight/normal weight participants than in those with overweight/obesity. The levels of IL‐8 varied over three orders of magnitude in both participant groups, with mean values of 285 ± 311 for underweight/normal participants and 222 ± 301 pg/ml for participants with overweight/obesity. Therefore, IL‐8 was excluded from further analysis.

**TABLE 4:** Association between each obesity index with leptin and inflammatory markers with each obesity index, adjusted for age group, sex, education level of the head of household, and area of residence and IDDS (models 1–4; a–c)

Dependent variable: Leptin and inflammatory markers	*N*	Quartiles	Weight status (BMI) (overweight/obesity vs. normal weight) OR (95%)	Waist circumference (high vs. normal) OR (95%)	Body fat % (high vs. normal) OR (95%)
Models			(a)	(b)	(c)
1. Leptin (*N* = 440)	113	First quartile	Ref	Ref	Ref
	112	Second quartile	**7.73 (1.64; 36.45)**	1.90 (0.67; 5.17)	**5.80 (1.79; 18.75)**
	114	Third quartile	**28.61 (6.38; 128.3)**	**7.73 (2.97; 20.11)**	**22.01 (6.97; 69.50)**
	101	Fourth quartile	**73.02 (16.00; 333.2)**	**21.55(7.99; 58.09)**	**87.19 (25.80; 294.7)**
2. CRP (*N* = 482)	130	First quartile	Ref	Ref	Ref
	126	Second quartile	**2.31 (1.18; 4.55)**	1.92 (0.95; 3.89)	**2.27 (1.18; 6.44)**
	129	Third quartile	**2.42 (1.23; 4.78)**	**2.30 (1.14; 4.62)**	**3.41 (1.77; 6.55)**
	97	Fourth quartile	**5.25 (2.57; 10.74)**	**4.03 (1.93; 8.45)**	**4.97 (2.46; 10.05)**
3. IL‐6 (*N* = 420)	114	First quartile	Ref	Ref	Ref
	105	Second quartile	0.72 (0.37; 1.41)	1.24 (0.60; 2.53)	0.95 (0.49; 1.83)
	107	Third quartile	0.82 (0.43; 1.59)	1.38 (0.68; 2.81)	1.17 (0.61; 2.25)
	94	Fourth quartile	0.67 (0.34; 1.34)	0.96 (0.45; 2.04)	0.84 (0.42; 1.67)
4. TNF‐α (*N* = 408)	108	First quartile	Ref	Ref	Ref
	105	Second quartile	1.59 (0.81; 3.12)	1.19 (0.58; 2.47)	1.11 (0.57; 2.15)
	99	Third quartile	1.26 (0.63; 2.53)	0.88 (0.42; 1.84)	0.92 (0.46; 1.82)
	96	Fourth quartile	0.92 (0.45; 1.87)	1.01 (0.48; 2.13)	0.86 (0.43; 1.71)

*Note*: The bold values indicate statistically significant (*p*‐value < 0.05).

Abbreviations: BMI, body mass index; CRP, C‐reactive protein; IDDS, individual dietary diversity score; IL‐6, interleukin‐6; TNF‐α, tumor necrosis factor.

The majority of participants with overweight and obesity were aged 18–45 years (49.1% and 61.5%, respectively) and above 45 years (43.6% and 38.5%, respectively). The proportion of participants with Leptin and CRP levels in the fourth quartile was highest among those with obesity (Table 2). Pearson correlation results showed that leptin was positively correlated with CRP (*r* = 0.098, *p* = 0.040) (Table 3).

**TABLE 5:** Multivariate association between three obesity indices with leptin and inflammatory markers with all three obesity indices combined, adjusted by age group, sex, education level of the head of household, area of residence models, and IDDS (models 5–8)

Dependent variable: Leptin and inflammatory markers	*N*	Quartiles	Weight status (BMI) (overweight/obesity vs. normal weight) OR (95%)	Waist circumference (high vs. normal) OR (95%)	Body fat % (high vs. normal) OR (95%)
Models			(a)	(b)	(c)
5. Leptin (*N* = 440)	113	First quartile	Ref	Ref	Ref
	112	Second quartile	3.34 (0.54; 20.60)	1.01 (0.33; 3.07)	3.14 (0.78; 12.71)
	114	Third quartile	**5.75 (1.00; 33.03)**	2.64 (0.90; 7.70)	**6.24 (1.60; 24.26)**
	101	Fourth quartile	**6.36 (1.09; 37.12)**	**4.87 (1.59; 14.94)**	**19.23 (4.70; 78.66)**
6. CRP (*N* = 482)	130	First quartile	Ref	Ref	Ref
	126	Second quartile	1.49 (0.52; 4.27)	1.20 (0.52; 2.78)	1.56 (0.56; 4.29)
	129	Third quartile	0.83 (0.29; 2.32)	1.25 (0.54; 2.88)	**3.49 (1.31; 9.31)**
	97	Fourth quartile	2.33 (0.76; 7.18)	1.72 (0.71; 4.17)	2.02 (0.67; 6.13)
7. IL‐6 (*N* = 420)	114	First quartile	Ref	Ref	Ref
	105	Second quartile	0.43 (0.14; 1.32)	1.58 (0.67; 3.74)	1.46 (0.48; 4.45)
	107	Third quartile	0.40 (0.14; 1.19)	1.58 (0.67; 3.73)	1.89 (0.64; 5.6)
	94	Fourth quartile	0.50 (0.16; 1.51)	1.20 (0.49; 2.98)	1.34 (0.44; 4.13)
8. TNF‐α (*N* = 408)	108	First quartile	Ref	Ref	Ref
	105	Second quartile	2.56 (0.85; 7.68)	1.02 (0.42; 2.47)	0.54 (0.18; 1.62)
	99	Third quartile	2.22 (0.71; 6.95)	0.80 (0.32; 1.96)	0.57 (0.18; 1.77)
	96	Fourth quartile	1.02 (0.34; 3.03)	1.16 (0.47; 2.88)	0.79 (0.27; 2.28)

*Note*: The bold values indicate statistically significant (*p*‐value < 0.05).

Abbreviations: BMI, body mass index; CRP, C‐reactive protein; IDDS, individual dietary diversity score; IL‐6, interleukin‐6; TNF‐α, tumor necrosis factor.

### 3.2 Association between leptin and inflammatory markers and obesity indices

Table 4 presents results of quasi‐linear association of single obesity indices with leptin and inflammatory markers, while Table 5 comprises results of obesity indices combined in one model for each outcome. In the separate models, individuals with obesity were more likely to fall in the higher quartiles of leptin and CRP, no association with IL‐6 and TNF‐α was found (Table 4). In particular, participants who were overweight or obese had a higher chance of having the highest levels of leptin (fourth quartile, model 1a; OR = 73.02 [95% CI, 16.00; 333.2]) and CRP (fourth quartile, model 2a; OR = 5.25 [95 % CI, 2.57; 10.74]). However, these associations were not prominent for CRP quartiles when obesity indices were combined in one model, except for participants with high %BF, who were significantly more likely to have higher CRP levels (third quartile, Table 5, model 6; OR = 3.49 [95% CI, 1.31; 9.31]). Although they were considered simultaneously, obesity indices (BMI and %BF), were significantly associated with high levels of leptin (third and fourth quartiles) and WC only in the fourth quartile (Table 5, model 5).

## 4 DISCUSSION

In the present study, the association between obesity indices (BMI, WC, and %BF), leptin and low‐grade inflammation (CRP, IL‐6, TNF‐α) in a Zanzibari population of individuals aged 5 years and above was investigated. Similar to other studies,^12^, ^15^, ^24^, ^25^, ^26^ this study population is undergoing a coexistence of double burden of underweight children and adolescents below 18 years, and overweight/obese adults above 18 years.

The observed association in the individual models between overweight/obesity and leptin is in agreement with previous studies.^7^, ^27^, ^28^ Consistent with previous findings in a sub‐Saharan African population,^28^ increased serum leptin concentrations for high BMI, WC, or %BF were observed, whereas—regardless of weight—leptin levels seem to correlate with all adipose tissue depots.^27^ Leptin is a hormone predominantly produced in the white adipose tissue of the human body, and the amount of leptin circulating in the body is proportional to the amount of fat of an individual.^29^ Thus, the key factor influencing leptin concentrations in human is adipose tissue mass.

Similar to other studies,^30^, ^31^, ^32^ this study population reported a significant association between overweight/obesity and elevated CRP levels. However, the possibility of obesity‐related comorbidities such as hypertension, diabetes, and probably high infection contributing to elevated CRP levels, could not be excluded. This study population included healthy (nonhospitalized) participants; hence the elevated CRP levels are more likely to be linked to adiposity. High %BF in the combined models of the present study was significantly linked to high CRP levels in the third quartile. The lack of significance in the highest quartile of %BF might have been due to the small sample size of participants in this particular quartile. Nevertheless, %BF proved to be the strongest correlate of CRP in this study population, affirming that high body fat plays an important part in stimulating increased levels of circulating CRP.^33^ Similar results were reported by Forouhi et al., who investigated the association between CRP and %BF in Europeans and South Asians. In the study, %BF and BMI showed a significant association with CRP in Europeans, while visceral fat and waist girth were strongly associated with CRP levels in South Asians.^34^

Furthermore, a US study reported significant associations between CRP and BMI of young adults,^31^ while studies from Tunisia^35^ and from Teheran^3^ reported associations between high WC and CRP. In this study, high %BF was observed to be the strongest correlate for CRP, compared to high BMI and high WC. This is probably due to the higher proportion of younger (5–18 years) underweight participants in the Zanzibari data set compared to the older populations in the Tunisian (35–70 years)^35^ and the US third NHANES wave (17–39 years)^31^ studies referred to above. Furthermore, the fact that the proportion of severe thinness and thinness participants for the CRP levels in the fourth quartile in our study was higher than that in the normal weight group, leads to the assumption that the higher CRP levels in the underweight participants were due to other causes such as a weak immune system/infection. This would then have attenuated the association between BMI/WC and CRP in the overall sample. Nevertheless, our findings show that %BF may serve as an indicator for obesity‐related increase of CRP release and insulin resistance in the study population. In summary, the findings from the previous studies^3^, ^31^, ^34^, ^35^ and the observations reported in the current study confirm strong association between obesity indices and CRP.

Overall, a positive correlation was observed between serum concentration of leptin and CRP independent of the obesity indices, as reported previously.^36^ This can easily be explained by the proinflammatory effect of leptin, which induces the release of CRP by hepatocytes.^37^ In turn, CRP leads to leptin resistance by interacting with the leptin receptor at a site distinct from the leptin binding site.^38^ Besides leptin, IL‐6 is secreted by adipocytes and stimulates the synthesis of CRP and other acute‐phase proteins.^36^ Therefore, the levels of several cytokines in the study population were also explored.

Previous studies have reported associations between obesity and high IL‐8 concentrations^39^ secreted by adipocytes.^40^ For this reason, IL‐8 was also considered an outcome of interest in our study population. However, extremely high IL‐8 values were observed (up to more than 1000 pg/ml, mean 269 ± 309 pg/ml), leading to the exclusion of the participants concerned from the final regression models. This was done to avoid misinterpretation due to bacterial and/or viral infections, which might have affected the expression level of other inflammatory markers.

The overall mean IL‐6 concentration in the present study (2.9 pg/ml) was slightly higher than that of males (2.5 pg/ml) and females (2.3 pg/ml) reported in a West African (Ghana and Nigeria) study population,^32^ but lower than the cut‐off of 5 pg/ml, which was considered as high IL‐6 concentration in a Tanzanian study.^41^ The fact that the West African population was more urbanized than the current population, and thus strongly associated to low‐grade inflammation, might explain the difference in findings. The current population was masked by high inflammation rate, thus no association was found with IL‐6. However, IL‐6 showed no significant association with any of the obesity markers in the present study, which is contradictory to other studies.^3^, ^7^ Like leptin, IL‐6 and TNF‐α are produced and released from human adipocytes, and their elevated levels have been found to be strongly associated with all measures of obesity in other studies from Europe or the United States.^42^, ^43^

Our unexpected results regarding the higher mean concentration of IL‐6 and TNF‐α in underweight/normal weight participants and the fact that no significant association was observed in overweight/obese participants may be due to underlying causes (infection‐weak immune system) or malnutrition,^44^ which were not investigated in this study. It is important to note that in the Zanzibari population, such an effect is possibly obscured by an increased secretion of IL‐6 and TNF‐α from other sources as regulators of the immune system. Thus, likely to be more challenged in this population compared to populations in high income countries.

The major strength of this study is that it represents the first population‐based study in Zanzibar, Tanzania, enrolling nonhospitalized individuals to investigate the association of three different obesity‐related indices with leptin and different inflammatory markers in a sample including children from 5 years, adolescents, younger, middle age, and older adults.

The random selection of the study participants, the use of standardized anthropometrical and laboratory measurements, and the exclusion of participants with extreme values of leptin and inflammatory markers/indications of infection enhanced the quality of the data used and the statistical analyses. The overall sample size in this study was similar to other studies,^3^ but advantageously involved a wide range of age groups, although limiting the availability of data in single age groups, particularly in the group above 45 years (*N* = 77, 21.4 %). The overall study was powered to investigate the prevalence of malnutrition in the Zanzibari population and resulted in a higher sample size compared to the current sample size used for this analysis. While we acknowledge that the decreased sample size of the current analysis is a limitation that may lessen the scope of the results obtained, we are convinced that our results contribute to the literature of sub‐Saharan African studies and provide insight into associations in developing economies with tropical climates. This study is further limited by the cross‐sectional design, which may not accurately reflect longitudinal associations between changes in body fat and the long‐term inflammation status of the study population.

## 5 CONCLUSION

Adipocytes dysfunction—due to adipose expansion—may have local or systemic effects on inflammatory responses, which may then contribute to the initiation and progression of obesity‐induced metabolic and cardiovascular risk factors, such as type 2 diabetes mellitus. High %BF in this population was associated with higher leptin and higher CRP concentrations, which have been closely linked to insulin resistance, a known risk factor for noncommunicable diseases. The results of this study add important information for the development of obesity prevention interventions promoting healthy diets and healthy lifestyles that will consequently help to reduce cardiovascular risk in this study population.

This study contributes to the general understanding on the association between obesity—measured as fat mass—, leptin and low‐grade inflammation in the sub‐Saharan African population. Additionally, the results provide important information for public health stakeholders, policy makers, and researchers in similar contexts.

## CONFLICT OF INTEREST

The authors declare no conflict of interest.

## AUTHOR CONTRIBUTIONS

This manuscript represents original work that has not been published previously and is currently not considered by another journal. The authors' responsibilities were as follows: Antje Hebestreit and Maria Adam Nyangasa developed the idea for this manuscript and analysis strategy. Maria Adam Nyangasa and Christoph Buck conducted statistical analyses interpreted the data; Maria Adam Nyangasa wrote the manuscript and had primary responsibility for final content and submitting the manuscript for publication; Maria Adam Nyangasa, Antje Hebestreit, Christoph Buck, and Soerge Kelm revised the manuscript critically for important intellectual content; Maria Adam Nyangasa, Christoph Buck, Soerge Kelm, Mohammed Ali Sheikh, Kathrin Günther, and Antje Hebestreit were responsible for revisions and final approval of the manuscript.

## References

[1] DeBoer MD . Obesity, systemic inflammation, and increased risk for cardiovascular disease and diabetes among adolescents: a need for screening tools to target interventions. Nutrition. 2013;29(2):379‐386. 10.1016/j.nut.2012.07.003. 23022122 PMC3578702

[2] Greenberg AS , Obin MS . Obesity and the role of adipose tissue in inflammation and metabolism. Am J Clin Nutr. 2006;83(2):461S‐465S. 16470013 10.1093/ajcn/83.2.461S

[3] Faam B , Zarkesh M , Daneshpour MS , Azizi F , Hedayati M . The association between inflammatory markers and obesity‐related factors in Tehranian adults: Tehran lipid and glucose study. Iran J Basic Med Sci. 2014;17(8):577‐582. 25422750 PMC4240791

[4] Yamauchi T , Kamon J , Waki H , et al. The mechanisms by which both heterozygous peroxisome proliferator‐activated receptor gamma (PPARgamma) deficiency and PPARgamma agonist improve insulin resistance. J Biol Chem. 2001;276(44):41245‐41254. 10.1074/jbc.M103241200. 11533050

[5] Ouchi N , Parker JL , Lugus JJ , Walsh K . Adipokines in inflammation and metabolic disease. Nat Rev Immunol. 2011;11(2):85‐97. 10.1038/nri2921. 21252989 PMC3518031

[6] Omer MA , Ali EA . Relationship between Hba1c levels and inflammatory biomarkers (C‐reactive protein, Il6 and Tnf‐alpha) among type 2 diabetes mellitus‐Khartoum‐Sudan. Int J Med Biomed Studies. 2019;3(3). 10.32553/ijmbs.

[7] Marques‐Vidal P , Bochud M , Bastardot F , et al. Association between inflammatory and obesity markers in a Swiss population‐based sample (CoLaus Study). Obes Facts. 2012;5(5):734‐744. 10.1159/000345045. 23108472

[8] Osegbe I , Okpara H , Azinge E . Relationship between serum leptin and insulin resistance among obese Nigerian women. Ann Afr Med. 2016;15(1):14‐19. 26857932 10.4103/1596-3519.158524 PMC5452686

[9] Oshodi T , Ebuehi OA , Ojewunmi O , Udenze I , Soriyan T . Circulating adipokine levels in type 2 diabetes mellitus in Lagos, Nigeria. Nig Q J Hosp Med. 2012;22(1):25‐28. 23175876

[10] Ministry of Health Zanzibar . Zanzibar National NCD Risk Factor Survey 2011. Zanzibar, Tanzania: Ministry of Health; 2011. https://41.73.201.42/hmisnews/?cat=12.

[11] Tanzania National Bureau of Statistics (NBS) and ICF Macro . Tanzania Demographic and Health Survey 2010. Dar es Salaam, Tanzania: NBS and ICF Macro; 2011.

[12] Nyangasa MA , Kelm S , Sheikh MA . Hebestreit A . Design, response rates, and population: characteristics of a cross‐sectional study in Zanzibar, Tanzania. JMIR Res Protoc. 2016;5(4):e235. 10.2196/resprot. 27908845 PMC5159612

[13] Nyangasa MA , Hebestreit A , Kelm S , Sheikh MA , Buck C . Food access and Socio‐demographic correlates determine food consumption and food security in Zanzibari households. Int J Environ Res Public Health. 2019;16(9):1557. 10.3390/ijerph16091557 PMC6539455 31060201

[14] United Nations Educational Scientific and Cultural Organization (UNESCO) . International Standard Classification of Education. UNESCO Institute for statistics. H3C 3J7, Canada. 2011.

[15] Wells JC , Sawaya AL , Wibaek R , et al. The double burden of malnutrition: aetiological pathways and consequences for health. Lancet. 2020;395(10217):75‐88. 10.1016/s0140-6736(19)32472-9. 31852605 PMC7613491

[16] Kennedy G , Ballard T , Dop M , Food and Agriculture Organization of the United Nations (FAO) . Guidelines for Measuring Household and Individual Dietary Diversity. Rome, Italy: Food and Agriculture Organization of the United Nations (FAO); 2011; ISBN 978‐92‐5‐106749‐9. http://www.fao.org/3/i1983e/i1983e.pdf.

[17] Cole TJ , Lobstein T . Extended international (IOTF) body mass index cut‐offs for thinness, overweight and obesity. Pediatr Obes. 2012;7(4):284‐294. 10.1111/j.2047-6310.2012.00064.x. 22715120

[18] World Health Organization (WHO) . BMI for Age—5 to 19 Years (Z‐scores). Geneva, Switzerland: World Health Organization; 2007. https://www.who.int/growthref/who2007_bmi_for_age/en/.

[19] World Health Organization (WHO) . The International Classification of Adult Underweight, Overweight and Obesity According to BMI. Geneva, Switzerland: Adapted from WHO, 1995, WHO, 2000 and WHO 2004.

[20] Nagy P , Kovacs E , Moreno LA , et al. Percentile reference values for anthropometric body composition indices in European children from the IDEFICS study. Int J Obes. 2014;38(Suppl 2):S15‐S25. 10.1038/ijo.2014.131 25219408

[21] International Diabetes Federation (IDF) . The IDF Consensus Definition of the Metabolic Syndrome in Children and Adolescents. Brussels, Belgium: IDF Communications; 2007. https://idf.org/our-activities/advocacy-awareness/resources-and-tools/61:idf-consensus-definition-of-metabolic-syndrome-in-children-and-adolescents.html.

[22] McCarthy HD , Cole TJ , Fry T , Jebb SA , Prentice AM . Body fat reference curves for children. Int J Obes. 2006;30(4):598‐602. 10.1038/sj.ijo.0803232 16570089

[23] Gallagher D , Heymsfield SB , Heo M , Jebb SA , Murgatroyd PR , Sakamoto Y . Healthy percentage body fat ranges: an approach for developing guidelines based on body mass index. Am J Clin Nutr. 2000;72(3):694‐701. 10.1186/s12944-016-0264-x. 10966886

[24] Oddo VM , Rah JH , Semba RD , et al. Predictors of maternal and child double burden of malnutrition in rural Indonesia and Bangladesh. Am J Clin Nutr. 2012;95(4):951–958. 10.3945/ajcn.111.026070 22357721

[25] Food and Agriculture Organization of the United Nations (FAO) . The double burden of malnutrition—Case studies from six developing countries. FAO Food Nutr Pap. 2006;84:1–334. https://pubmed.ncbi.nlm.nih.gov/19172986/. 19172986

[26] Nyangasa MA , Buck C , Kelm S , Sheikh MA , Brackmann KL , Hebestreit A . Association between cardiometabolic risk factors and body mass index, waist circumferences and body fat in a Zanzibari cross‐sectional study. BMJ Open. 2019;9(7):e025397. 10.1136/bmjopen-2018-025397. PMC6615808 31278089

[27] Haffner SM , Gingerich RL , Miettinen H , Stern MP . Leptin concentrations in relation to overall adiposity and regional body fat distribution in Mexican Americans. Int J Obes Relat Metab Disord. 1996;20(10):904‐908. 8910093

[28] Ayina CNA , Noubiap JJN , Etoundi Ngoa LS , et al. Association of serum leptin and adiponectin with anthropomorphic indices of obesity, blood lipids and insulin resistance in a sub‐Saharan African population. Lipids Health Dis. 2016;15(1):96. 10.1186/s12944-016-0264-x. 27189377 PMC4869296

[29] Clement K , Vaisse C , Lahlou N , et al. A mutation in the human leptin receptor gene causes obesity and pituitary dysfunction. Nature. 1998;392(6674):398‐401. 10.1038/32911. 9537324

[30] Choi J , Joseph L , Pilote L . Obesity and C‐reactive protein in various populations: a systematic review and meta‐analysis. Obes Rev. 2013;14(3):232‐244. 10.1111/obr.12003. 23171381

[31] Visser M , Bouter LM , McQuillan GM , Wener MH , Harris TB . Elevated C‐reactive protein levels in overweight and obese adults. J Am Med Assoc. 1999;282(22):2131‐2135. 10.1001/jama.282.22.2131 10591334

[32] Doumatey AP , Lashley KS , Huang H , et al. Relationships among obesity, inflammation, and insulin resistance in African Americans and West Africans. Obesity (Silver Spring), 2010;18(3):598‐603. 10.1038/oby.2009.322. 19798069 PMC4151268

[33] Anty R , Bekri S , Luciani N , et al. The inflammatory C‐reactive protein is increased in both liver and adipose tissue in severely obese patients independently from metabolic syndrome, Type 2 diabetes, and NASH. Am J Gastroenterol. 2006;101(8):1824‐1833. 10.1111/j.1572-0241.2006.00724.x. 16790033

[34] Forouhi NG , Sattar N , McKeigue PM . Relation of C‐reactive protein to body fat distribution and features of the metabolic syndrome in Europeans and South Asians. Int J Obes. 2001;25:1327. 10.1038/sj.ijo.0801723. 11571595

[35] Belfki H , Ben Ali S , Bougatef S , et al. Relationship of C‐reactive protein with components of the metabolic syndrome in a Tunisian population. Eur J Intern Med. 2012;23(1):e5‐e9. 10.1016/j.ejim.2011.10.011. 22153549

[36] Motie M , Evangelista LS , Horwich T , et al. Association between inflammatory biomarkers and adiposity in obese patients with heart failure and metabolic syndrome. Exp Ther Med. 2014;8(1):181‐186. 10.3892/etm.2014.1673. 24944619 PMC4061200

[37] Hribal ML , Fiorentino TV , Sesti G . Role of C reactive protein (CRP) in leptin resistance. Curr Pharm Des. 2014;20(4):609‐615. 10.2174/13816128113199990016. 23688010 PMC4155811

[38] Sudhakar M , Silambanan S , Chandran AS , Prabhakaran AA , Ramakrishnan R , C‐Reactive Protein (CRP) and Leptin Receptor in Obesity . Binding of monomeric CRP to leptin receptor. Front Immunol. 2018;9:1167. 10.3389/fimmu.2018.01167. 29910808 PMC5992430

[39] Straczkowski M , Dzienis‐Straczkowska S , Stepien A , Kowalska I , Szelachowska M , Kinalska I . Plasma interleukin‐8 concentrations are increased in obese subjects and related to fat mass and tumor necrosis factor‐alpha system. J Clin Endocrinol Metab. 2002;87(10):4602‐4606. 10.1210/jc.2002-020135. 12364441

[40] Bruun JM , Pedersen SB , Richelsen B . Regulation of interleukin 8 production and gene expression in human adipose tissue in vitro. J Clin Endocrinol Metab. 2001;86(3):1267‐1273. 10.1210/jcem.86.3.7264. 11238519

[41] Nkinda L , Patel K , Njuguna B , et al. C‐reactive protein and interleukin‐6 levels among human immunodeficiency virus ‐infected patients with dysglycemia in Tanzania. BMC Endocr Disord. 2019;19(1):77. 10.1186/s12902-019-0407-y. 31331321 PMC6647154

[42] Popko K , Gorska E , Stelmaszczyk‐Emmel A , et al. Proinflammatory cytokines Il‐6 and TNF‐alpha and the development of inflammation in obese subjects. Eur J Med Res. 2010;15(Suppl 2),:120–122. 21147638 10.1186/2047-783X-15-S2-120 PMC4360270

[43] Kern PA , Ranganathan S , Li C , Wood L , Ranganathan G . Adipose tissue tumor necrosis factor and interleukin‐6 expression in human obesity and insulin resistance. Am J Physiol Endocrinol Metab. 2001;280(5):E745‐E751. 10.1152/ajpendo.2001.280.5.E745. 11287357

[44] Songür N , Kuru B , Kalkan F , Ozdilekcan C , Cakmak H , Hizel N . Serum interleukin‐6 levels correlate with malnutrition and survival in patients with advanced non‐small cell lung cancer. Tumori. 2004;90(2):196‐200. 15237582 10.1177/030089160409000207

